# Study on the Effect of Gamma-Ray Irradiation on the Adsorption of ^99^Tc and Re by a Silica-Based Pyridine Resin

**DOI:** 10.3390/toxics10110638

**Published:** 2022-10-25

**Authors:** Hongji Sang, Cong Mao, Yan Wu, Yuezhou Wei

**Affiliations:** School of Nuclear Science and Engineering, Shanghai Jiao Tong University, Shanghai 200240, China

**Keywords:** ^99^Tc, adsorption, pyridine resin, γ-ray absorbed doses

## Abstract

A silica-based anion exchange resin was synthesized and used to remove ^99^Tc from real radioactive liquid waste. The adsorbent had a uniform particle size and exhibited good thermal stability up to 100 °C, which is promising for large-scale column experiments. In accordance with the chemical similarity with Tc, Re was used as a surrogate in this study. The N 1s high-resolution XPS spectra of the adsorbent before and after the adsorption of Re indicated that the ion exchange reaction was the controlling mechanism in the process. After γ-ray irradiation, the changing trend of the *K*_d_ was consistent, which showed that the competitive adsorption of NO_3_^−^ led to a decrease in *K*_d_. The adsorption capacity for the Re decreased slightly from 35.8 to 31.9 mg/g with the increase in the absorbed dose from 0 to 50 kGy. The separation and recovery of Re and the coexisting ions were achieved by chromatographic separation experiments, and the recovery percentage of Re was 86%. In real radioactive liquid waste, N3/SiO_2_ exhibited good selectivity toward ^99^Tc over the coexisting metals, namely, ^90^Sr, ^137^Cs, ^241^Am, and U, and the decontamination efficiency of ^99^Tc attained 65%.

## 1. Introduction

The removal of long-lived nuclides from high-level liquid waste (HLLW) could improve the safety of the spent fuel reprocessing and waste disposal process [1]. The radionuclide ^99^Tc, with a high yield and a long half-life, is generated by the uranium fission reaction, and it is a hazardous nuclide commonly found in HLLW [2,3,4]. The behavior of Tc in the environment is influenced by biochemical processes and is therefore very complex. The pertechnetate anion (TcO_4_^−^) is the main existence form for ^99^Tc in radioactive wastewater, which has a high solubility and mobility in ground water and soil, posing a potential threat to human health [5,6,7,8]. In addition, technetium metal can be used to prepare β radiation sources of low neutron and superconductors, while TcO_4_^−^ can improve the corrosion resistance of steel [9]. Note that technetium is mostly considered to be a dangerous waste at present due to its environmental mobility and long half-life. To protect the environment and human health, the separation and recovery of ^99^Tc from HLLW is highly valuable [9,10,11]. However, the effective removal of ^99^Tc from wastewater has always been challenging. Furthermore, in the absence of an electron transfer reaction, Re is usually used as a non-radioactive analogue for ^99^Tc because of their similar geochemical properties and ion size [2,12,13].

Under the plutonium and uranium redox extraction (PUREX) conditions, technetium can coextract with other species in solution, including zirconium and uranium [14]. The separation of technetium usually requires additional processes. Moreover, a large amount of secondary organic waste is generated, which increases the cost of the reprocessing process. The extraction behavior of technetium is complex due to the versatile chemical properties, so efficiency extractants with good selectivity need to be developed. With the characteristics of large exchange capacity, excellent selectivity, low cost, and less waste accumulation [15,16,17], ion exchange as an environmentally friendly technology has become an optimal choice. Until now, various ion exchangers including thorium borates [18], layered double hydroxides (LDHs) [19], metal sulfides [20], poly-electrolytes [15] and ion exchange resins [9,21,22,23,24,25,26] have been studied for the selective separation of ^99^Tc. Among these, anion exchange resin with amine groups is one kind of promising material, which can capture negatively charged TcO_4_^−^ in an aqueous solution by an anion exchange reaction. Our group prepared a pyridine type weakly basic anion exchange resin through copolymerization without chloromethylation and amination [27], simplifying the synthesis process of resins.

**Figure 1:**
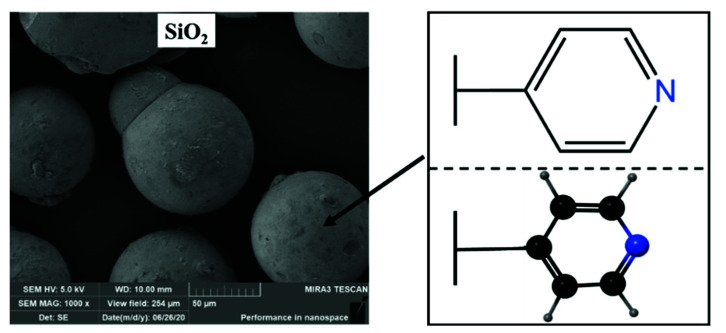
Schematic representation of the N3/SiO_2_ structure.

In this study, the pyridine type weakly basic anion exchange resin (N3), with high affinity to ^99^Tc, was impregnated as the adsorbent into the pores of a rigid support material SiO_2_ (N3/SiO_2_) to enhance the hydraulic properties and radiation stability (Figure 1). The effect of the γ-ray, nitric acid concentration, initial metal concentration, and coexisting ions on the adsorption performance toward technetium and its analogue (rhenium) was investigated. The chromatographic separation was performed by dynamic column, and the selective separation of ^99^Tc from real radioactive liquid waste was evaluated.

## 2. Materials and Methods

### 2.1. Materials

The chemical reagents α,α′-azobisisobutyronitrile (AIBN) with chemical pure purity, 1,1′-azobiscyclohexane-1-carbonitrile (ACCN) with 98% purity, 4-vinylpyridine (4 VP) with 95% purity, tert-butyl-catechol (TBC) with 98% purity and divinylbenzene (DVB) with 80% purity were purchased from Beijing InnoChem Science & Technology Co., Ltd., Beijing, China. Ammonium rhenate (NH_4_ReO_4_), ammonium chloride (NH_4_Cl), molybdenum pentachloride (MoCl_5_), sodium hydroxide (NaOH), thiourea, methanol, acetophenone, diethyl-o-phthalate, hydrochloric acid, nitric acid, and the other nitrates were from Sinopharm Chemical Reagent Co., Ltd., Shanghai, China, and they were of analytical grade.

### 2.2. Preparation of the N3/SiO_2_ Adsorbent

The N3/SiO_2_ was prepared as follows. The SiO_2_ was washed three times with concentrated hydrochloric acid and methanol, respectively, to remove the metal impurities and increase its activity. After filtration, the SiO_2_ was dried at 90 °C for 24 h. Then, the SiO_2_ was placed in a flask of a rotary evaporator, and a mixture of initiators (AIBN and ACCN), diluents (acetophenone and diethyl-o-phthalate), and monomers (4 VP and DVB) was sucked into the abovementioned flask under the action of a vacuum pump. The flask was gradually heated to 90 °C and rotated slowly. During the process, the monomers after successful polymerization were impregnated into the pores of SiO_2_ under the protection of the nitrogen atmosphere [27]. Finally, the product was washed and dried.

### 2.3. Irradiation Experiments

N3/SiO_2_ was irradiated at room temperature using a ^60^Co source with an irradiation dose rate of 5 kGy/h. Then, batch adsorption experiments were carried out with the N3/SiO_2_ after irradiation in different concentrations of nitric acid solution.

### 2.4. Characterization

A laser diffraction particle size analyzer (S3500, Microtrac Inc., Largo, FL, USA) was used to measure the particle-size distribution characteristics of the N3/SiO_2_. The average pore size and pore size distribution were tested by the mercury intrusion method (AutoPore IV 9500 V1.09, Micromeritics Inc., Norcross, GA, USA). The thermal stability of the N3/SiO_2_ was examined by thermogravimetry–differential thermal analysis (DTG-60, Shimadzu Corporation, Kyoto, Japan) with a heating rate of 10 °C/min in an air atmosphere. The composition and valence states of the main elements were tested through X-ray photoelectron spectroscopy (XPS, AXIS Ultra DLD, Shimadzu Corporation, Kyoto, Japan).

### 2.5. Batch Experiments

In the batch experiments, 50 mg of the adsorbent and 5 mL of HNO_3_ solution were added to a glass vial with a plastic lid. The vial was shaken mechanically at 120 rpm and 25 °C for a certain contact time. The concentrations of metal ions in the aqueous phase were measured via atomic absorption spectrophotometry (AAS, SP3880, Shanghai Guangpu Co., Ltd., Shanghai, China) and sequential plasma spectrometry (ICP-AES, ICP-7510, Shimadzu Corporation, Kyoto, Japan). The distribution coefficients (*K*_d_) and adsorption amount (*Q*_eq_) of the metal ions were calculated by the following equations [28]:

(1) $$K_{d}=\frac{C_{0}-C_{e}}{C_{e}}\times \frac{V}{m},$$

(2) $$Q_{\mathrm{eq}}=\left(C_{0}-C_{e}\right)\times \frac{V}{m},$$

where *C*_0_ and *C*_e_ (mmol/L) represent the initial and equilibrium concentrations of metal ions in an aqueous phase, respectively. *V* (mL) and *m* (g) denote the volume of the aqueous phase and the weight of the adsorbent, respectively.

### 2.6. Chromatographic Partitioning of Re

The chromatographic partitioning of Re from simulated HLLW (SHLLW) containing typical fission and non-fission elements was conducted to evaluate the feasibility and practicability of the N3/SiO_2_ in a column system. The N3/SiO_2_ of 1 g was packed in the glass column (10 mm diameter × 50 mm long); the SHLLW with the nitric acid concentration of 1 M was then fed through the adsorbent-packed column at a flow rate of 0.5 mL/min. The concentrations of the target elements in the effluent were tested by ICP and AAS.

### 2.7. ^99^Tc Removal Performance

The adsorption property for the real radioactive waste was further tested. A certain amount of radioactive liquid waste was obtained from a batch of nuclear facilities in China, and its nitric acid concentration was adjusted to 1 and 3 mol/L respectively. After adjustment, the gross radioactivity of technetium used in this experiment was approximately 1 × 10^3^ Bq. The abovementioned liquid waste was mixed with N3/SiO_2_ and shaken mechanically for 12 h at 25 °C. The ratio of the solid phase to the aqueous phase was 0.5 g to 5 cm^3^. The amounts of ^90^Sr and ^99^Tc were determined by a liquid scintillation analyzer. An HPGe gamma spectrometer was used to measure the activities of ^137^Cs and ^241^Am.

## 3. Results and Discussion

### 3.1. Characterization

#### 3.1.1. Particle Size and Pore Size Analysis

**Figure 2:**
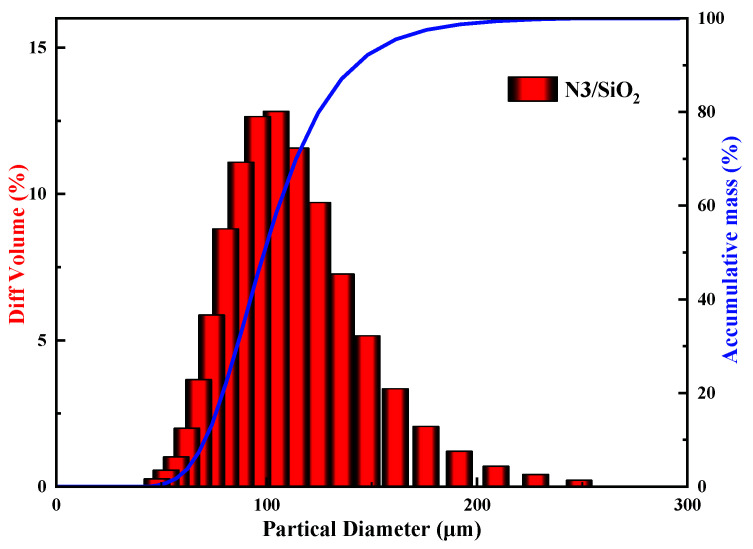
Particle size analysis curve of the N3/SiO_2_ adsorbent.

The particle-size distribution characteristics of the N3/SiO_2_ obtained by the laser particle size analyzer are shown in Figure 2. The cumulative curve showed that 80% of the particles were mainly distributed between 60 and 140 microns, with an average particle size of 114.9 microns. The separation efficiency was improved by reducing the back-mixing when the fluid passed through the adsorbent bed filled with the adsorbent of uniform particles.

**Figure 3:**
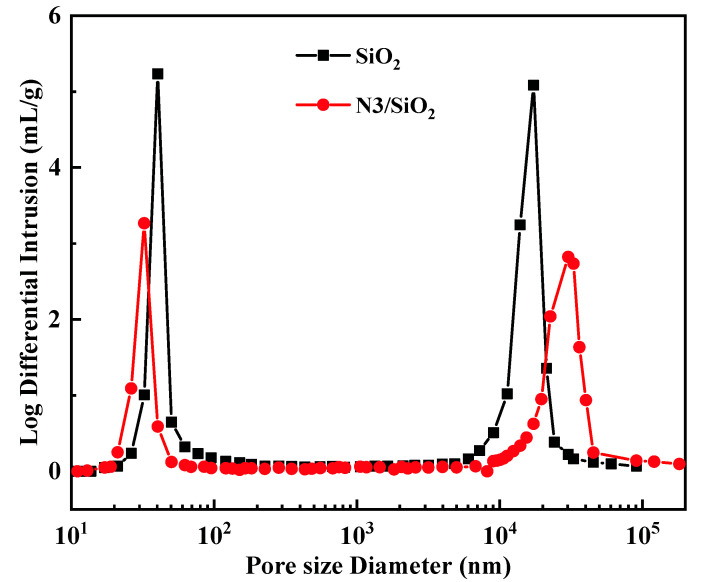
The pore size distribution of SiO_2_ and N3/SiO_2_.

The MIP method was used to analyze the porosity and pore size of the SiO_2_ and N3/SiO_2_. Figure 3 presents the pore size distribution of the SiO_2_ and N3/SiO_2_. The most probable pore size was located at the abscissa value of the peak. There was no significant difference in the pore-size distribution tendency between the two samples. The two peaks located at 10 to 50 μm were considered to arise from the interspace between the SiO_2_ particles. The pore capacity increment of the silica carrier was concentrated in the pore size range of 26–62 nm and reached its maximum value at about 40 nm. The pore size distribution with good uniformity was conducive to the diffusion and impregnation of the adsorbate into the SiO_2_ pores. The pore size of the SiO_2_ was calculated to be 43.3 nm, and it decreased to 34.8 nm after the pyridine resin was loaded (Table 1). Similarly, the porosity of the N3/SiO_2_ was evaluated at 70.7%, slightly lower than the SiO_2_. The impregnation of the pyridyl resin into the SiO_2_ pores resulted in a decrease in the pore size and porosity. Furthermore, a larger porosity was beneficial to improve the ion exchange rate in the adsorption process.

#### 3.1.2. Thermal Property

**Figure 4:**
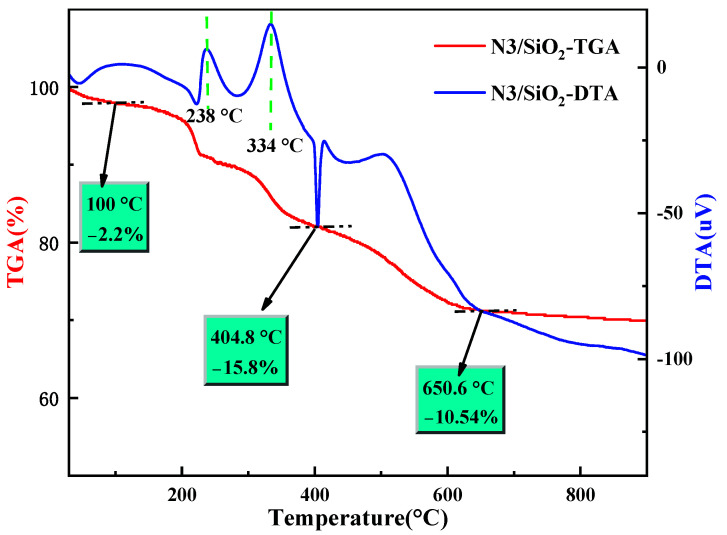
The TGA/DTA curves of the N3/SiO_2_.

As shown in Figure 4, the thermal decomposition behavior of N3/SiO_2_ was analyzed. The weight loss within 100 °C was 2.2 wt%, corresponding to the evaporation of water in the adsorbent. The continuous rapid weight loss starting at about 200 °C could be regarded as the decomposition of the resin polymer loaded into the SiO_2_ carrier. There were two obvious exothermic peaks in the DTA curves at about 238 °C and 334 °C, respectively. The N3 resin obtained by the polymerization of 4-vinylpyridine and divinylbenzene as monomers mainly contained two crosslinking structures, a pyridine ring and a benzene ring, which were connected to the skeleton structure; the two main structures of the resin polymer were decomposed successively under these two temperature conditions. The weight loss calculated at around 404.8 °C was inferred to be the thermal desorption of the self-polymers of a few monomers with a corresponding endothermic peak. The residue after 650.6 °C was the stable silica carrier. In summary, the N3/SiO_2_ lost approximately 28.54 wt% of its original weight at 30–900 °C, which indicated that 26.34 wt% of the organics were immobilized. All the results demonstrated the successful synthesis of the adsorbent, which exhibited good thermal stability under 100 °C.

#### 3.1.3. XPS

**Figure 5:**
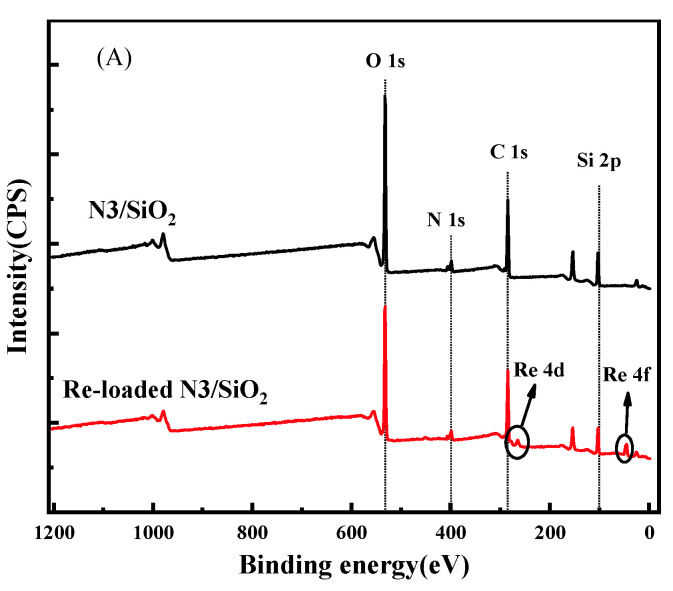

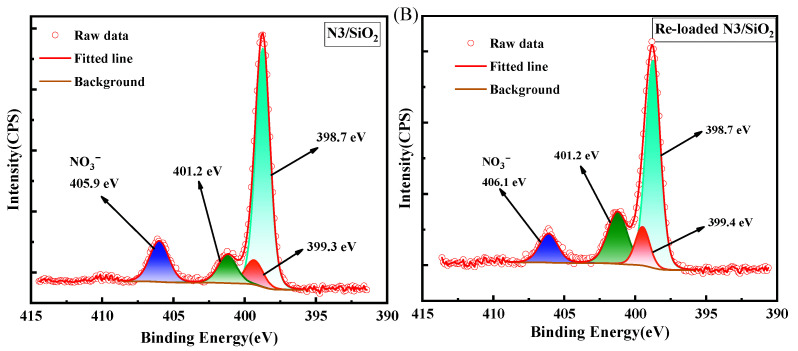
(**A**) Wide XPS spectra of N3/SiO_2_ and Re-loaded N3/SiO_2_; (**B**) High resolution XPS spectra of N 1s in N3/SiO_2_ and Re-loaded N3/SiO_2_.

As shown in Figure 5, XPS was used to investigate the mechanism of the adsorption of N3/SiO_2_ for Re. In Figure 5A, the peaks of O 1s, N 1s, C 1s, and Si 2p are observed in the full spectrum of the adsorbent before and after the Re adsorption. Moreover, new peaks at 264.62 eV and 45.37 eV were assigned to Re 4d and Re 4f, respectively, in the latter case, thereby confirming the successful adsorption of Re into the N3/SiO_2_. The N 1s spectra of the absorbent before and after the adsorption of Re was further analyzed, as shown in Figure 5B. The components of the N 1s spectrum before and after adsorption could be deconvoluted into four peaks, indicating that no new valence of N was produced in the adsorption reaction. The lowest binding energy peak centered at 398.7 eV was ascribed to the deprotonated N atoms in the pyridine functional group, whereas the 399.3 and 401.2 eV contributions were due to the N atoms in different degrees of H acceptance and protonation in the pyridine functional group [29,30]. Since the atomic orbital arrangement of the N element was 1S^2^2S^2^2P^3^, there was a lone pair of electrons outside its nucleus, which easily combined with hydrogen ions to form a stable state. Due to the inevitable differences in the degree of protonation of the adsorbent in the two cases, the intensities of the contribution in both cases were also different [30]. From the above results, the adsorption mechanism of the N3/SiO_2_ for Re mainly arose from the ion exchange reaction; that is, ReO_4_^−^ combined with the protonated nitrogen element on the pyridine functional group.

**Figure 6:**
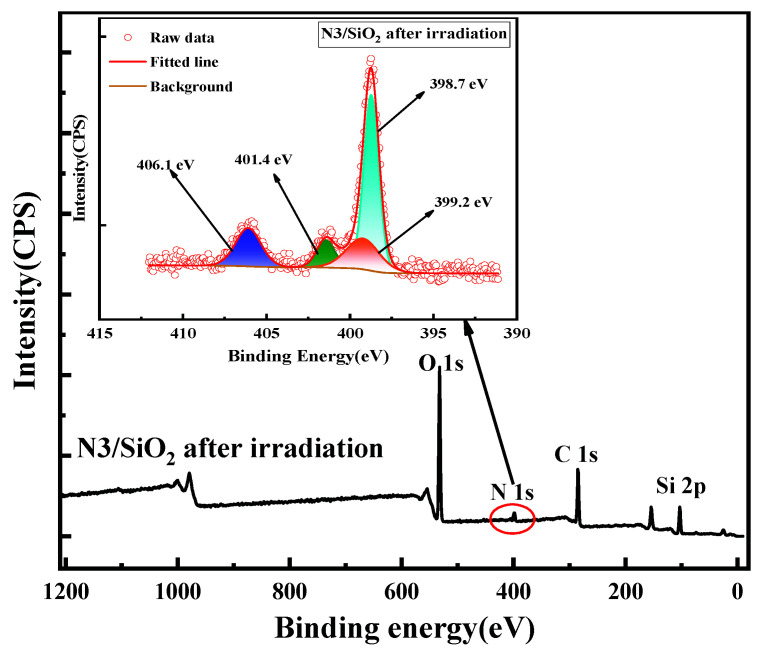
XPS spectra of the irradiated N3/SiO_2_.

The XPS spectrum of the N3/SiO_2_ with an absorbed dose of 100 kGy was further analyzed. In Figure 6, the N 1s spectrum of irradiated N3/SiO_2_ could be decomposed into four peaks, consistent with the fresh adsorbent. The areas of the two peaks near 399.2 eV and 401.4 changed greatly, which may be due to the gamma irradiation on the protonation process of the adsorbent.

### 3.2. Effects of the γ-ray Irradiation on the Re Adsorption in Nitric Acid

**Figure 7:**
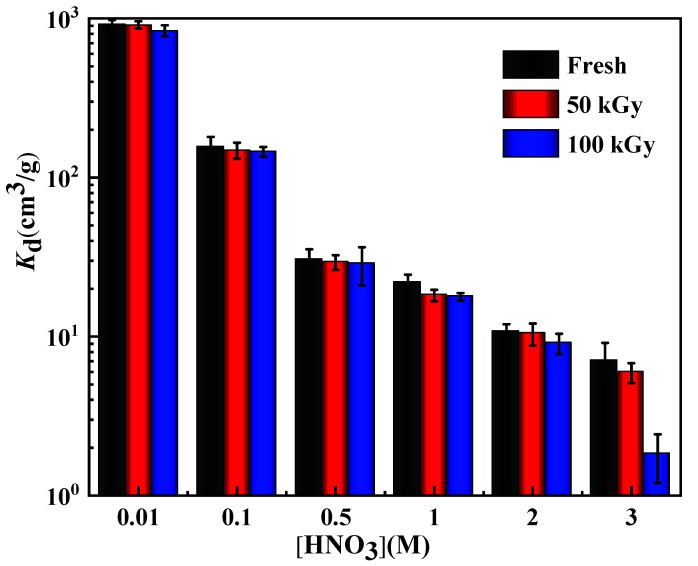
The effects of the γ-ray irradiation on different concentrations of nitric acid, with the following adsorption conditions: *V*/*m* = 100 cm^3^/g, [Re] =1 mM, T = 25 °C, and contact time = 12 h.

The effect of the HNO_3_ concentration on the *K*_d_ of the Re on the fresh and irradiated N3/SiO_2_ was studied by varying the HNO_3_ concentration in the range of 0.01–3 mol/L (Figure 7). At the absorbed dose of 50 and 100 kGy, the *K*_d_ of Re was consistent with the trend before irradiation; that is, it gradually decreased with the increase in the HNO_3_ concentration. This phenomenon of the decrease in the *K*_d_ value with an increasing concentration of HNO_3_ is explained by the competitive adsorption reaction of ReO_4_^−^ and NO_3_^−^ on the anion exchanger. Generally, the effect of nitric acid on the adsorption process in the adsorption environment depends on the strength of the two effects of salting-out and competition. As the concentration of HNO_3_ increased, its competitive adsorption gradually dominated, which reduced the electrostatic attraction to ReO_4_^−^, resulting in the weakening of the adsorption capacity for Re under this experimental condition. In addition, with the increase in the radiation-absorbed dose, the *K*_d_ of Re by the adsorbent gradually decreased. This result might be attributed to the influence of the gamma irradiation on the protonation of the adsorbent, and the gamma irradiation might also lead to structural changes in the resins. However, at the 100 kGy absorbed dose, the *K*_d_ value only decreased in the range of 5.6–18.4% when the concentration of HNO_3_ increased from 0.01 to 1 M, indicating an acceptable radiation-absorbed dose under 100 kGy. Considering the actual situation of the HLLW and the adsorption performance of the N3/SiO_2_ for Re, 1 M HNO_3_ was selected as the nitric acid condition for the later adsorption experiments.

### 3.3. Adsorption Isotherms of N3/SiO_2_ before and after Irradiation

**Figure 8:**
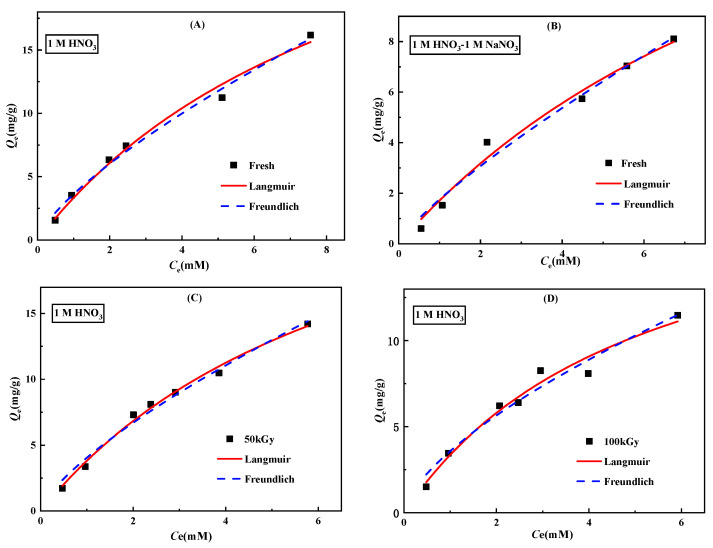
The adsorption isotherms of (**A**) the fresh N3/SiO_2_ in 1 M HNO_3_, (**B**) the fresh N3/SiO_2_ in a mixture of 1 M HNO_3_ and 1 M NaNO_3_, (**C**) the irradiated N3/SiO_2_ (50 kGy) in 1 M HNO_3_, and (**D**) the irradiated N3/SiO_2_ (100 kGy) in 1 M HNO_3_, with the following adsorption conditions: *V*/*m* = 100 cm^3^/g, T = 25 °C, and contact time = 12 h.

The adsorption capacity is a key parameter to evaluate the adsorption performance of the adsorbents. The experiments were carried out by changing the initial concentration of Re in the HNO_3_. As seen in Figure 8, the adsorption capacity increased gradually and then tended to be stable with the increase in the initial Re concentration. The adsorption isotherm can predict the interaction between the target ions and the active sites of the adsorbent. The adsorption behavior of the adsorbent before and after irradiation for Re was further analyzed using the classical Langmuir and Freundlich adsorption isotherm models. The Langmuir model assumes that the adsorption between the adsorbent and the target nuclide is a monolayer adsorption of finite sites. The equation is defined as follows [31,32]:

(3) $$Q_{e}=\frac{Q_{m}K_{L}C_{e}}{1+K_{L}C_{e}},$$

where *Q*_m_ is the maximum adsorption capacity (mg/g); *Q*_e_ indicates the equilibrium adsorption capacity (mg/g); *C*_e_ indicates the metal ion concentration in the aqueous phase (mmol/L); and *K*_L_ (L/g) represents the adsorption equilibrium constant.

The Freundlich adsorption isotherm model is usually applied to multilayer physical and chemical adsorption processes. The Freundlich adsorption isotherm equation is defined as follows [33,34,35]:

(4) $$Q_{e}=K_{F}C_{e}^{\frac{1}{n}},$$

where *K*_F_ is the adsorption capacity constant, and *n* is the adsorption intensity constant.

The considered adsorption isotherm models were used to fit the experimental data by a nonlinear regression method. Figure 8 shows the fitting curves, and Table 2 shows the fitting parameters of the adsorption isotherm model. As shown in Figure 8A,B, in 1 M HNO_3_ and a mixture of 1 M NaNO_3_ and 1 M HNO_3_, the maximum adsorption capacity for fresh N3/SiO_2_ obtained by the Langmuir isotherm model was 35.8 and 22.2 mg/g, respectively, which matched well with the experimental data. Further, the correlation constants of the Langmuir model in the two media (1 M HNO_3_ and a mixture of 1 M NaNO_3_ and 1 M HNO_3_) were greater than 0.98. The better fitting with the Langmuir model indicated that the homogeneous monolayer adsorption was the governing adsorption process. The difference in the adsorption capacity of the adsorbent in the two solutions was attributed to the competitive effect of the increased NO_3_^−^ in the solution on the adsorption of the ReO_4_^−^ by the N3/SiO_2_.

Figure 8C,D show the adsorption isotherm model fitting curves of N3/SiO_2_ to Re at different absorbed doses. It can be observed that as the absorbed dose increased from 50 to 100 kGy, the adsorption capacity obtained by the Langmuir model decreased from 31.9 to 20.9 mg/g, consistent with the results of the acidity experiment. The adsorption capacity of the N3/SiO_2_ was only 10.9% lower than that of fresh adsorbent at 50 kGy, indicating that the γ-ray irradiation had little effect on the absorption of Re at this condition.

### 3.4. Chromatographic Partitioning of Re

**Figure 9:**
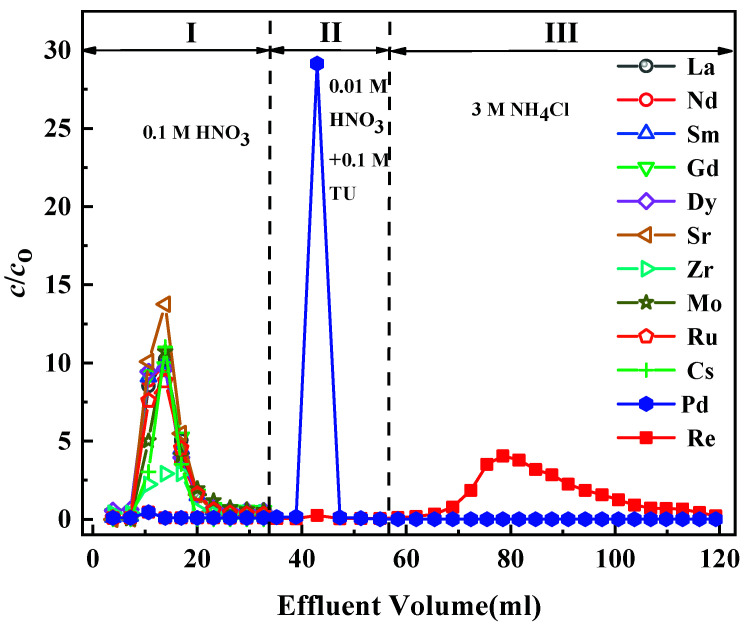
Chromatographic partitioning of Re from a simulated solution by the N3/SiO_2_ packed column, with the following adsorption conditions: [M] = 5 mmol/L, [HNO_3_] = 1 mol/L, and T = 25 °C.

To evaluate the separation of Re from the SHLLW in the column system, a feed solution containing 5 mmol/L Re(VII), La(III), Nd(III), Sm(III), Gd(III), Dy(III), Sr(II), Zr(IV), Mo(VI), Ru(III), Cs(I), and Pd(II) was passed through the N3/SiO_2_ packed column. Then, the given volumes of 0.1 M HNO_3_, 0.01 M HNO_3_–0.1 M Thiourea (TU), and 3 M NH_4_Cl were subsequently pumped into the column. As shown in Figure 9, except for Re(VII) and Pd(II), all coexisting ions exhibited no adsorption onto N3/SiO_2_ and flowed into the effluent along with the 0.1 mol/L HNO_3_ at stage I. In accordance with the adsorption behavior of La(III), Nd(III), Sm(III), and Gd(III), it could be inferred that almost all the trivalent RE(III) contained in HLLW would have no adverse effect on the separation of Re(VII) in a 1 mol/L HNO_3_ solution [36]. Unlike Re, the affinity of the adsorbent for Pd was a presumed complexation reaction; that is, the nitrogen atom with a lone pair of electrons on the pyridine functional group of the adsorbent could form a stable coordination bond with the empty orbit of the palladium atom [37,38]. The elution of Pd(II) by 0.01 M HNO_3_–0.1 M TU was effective, and the elution band appearing in the elution curve of Pd(II) was sharp and narrow, without elution tailing. This was ascribed to the strong complexation affinity of thiourea for Pd(II) [39]. Moreover, the presence of Re in the eluate could hardly be detected during the Pd(II) elution process (stage II). As 3 M NH_4_Cl was supplied to the column, the adsorbed Re(VII) on the N3/SiO_2_ was eluted efficiently (stage III). A high concentration of nitric acid is generally used as the eluent of anion exchange resins. Using 3 M NH_4_Cl as a substitute for concentrated nitric acid could not only improve the service life of the adsorbent but also reduce the corrosion of the equipment in practical applications. The calculated recovery percentage was approximately 86% for Re(VII) and 98% for Pd(II). The experimental results showed that the N3/SiO_2_ packed column could realize the selective separation and recovery of Re(VII) in SHLLW.

### 3.5. ^99^Tc Removal Performance

**Figure 10:**
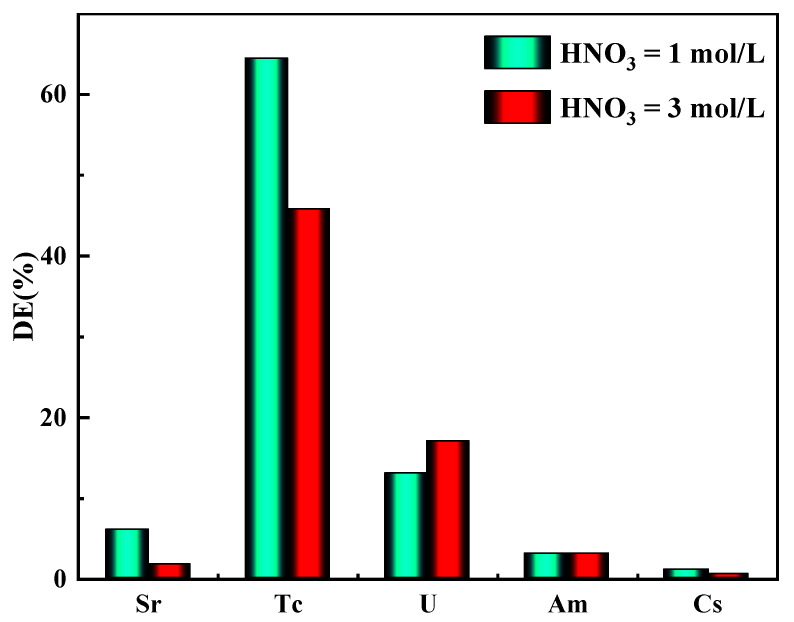
The decontamination efficiency of ^90^Sr, ^99^Tc, ^137^Cs, ^241^Am, and U for N3/SiO_2_ in radioactive liquid waste, with the following adsorption conditions: *V*/*m* = 10 cm^3^/g, T = 25 °C, and 12 h.

The decontamination efficiency (DE) of the adsorbent for ^90^Sr, ^99^Tc, ^137^Cs, ^241^Am, and U in radioactive liquid waste at 1 and 3 M HNO_3_ is shown in Figure 10. In the radioactive liquid waste of 1 M HNO_3_, the DE of ^99^Tc by the adsorbent reached 64.5%, and 45.8% in 3 M HNO_3_, much higher than those of the ^90^Sr, ^137^Cs, ^241^Am, and U. The DE of the N3/SiO_2_ for U was approximately 15% in both radioactive wastes. Uranyl nitrate is the main form of hexavalent uranium in nitric acid solution, and it is not easy to obtain ionic compounds using nitrite ions. Therefore, with the increase in the nitric acid concentration, the adsorption of U on N3/SiO_2_ did not change significantly. The adsorbent had basically no adsorption effect on the other nuclides, and the small amount of ^90^Sr, ^137^Cs, ^241^Am, and U attached to the adsorbent could be eluted by the dilute nitric acid to achieve the selective separation and recovery of ^99^Tc.

## 4. Conclusions

Re was selected as the analogue of radioactive ^99^Tc to carry out the batch adsorption experiments, and XPS analysis demonstrated that the adsorption of Re by the adsorbent was the result of the ion-exchange reaction. The adsorption experiment showed that the competitive adsorption produced by the high concentration of NO_3_^−^ inhibited the adsorption of ReO_4_^−^, resulting in the decrease in *K*_d_ with an increasing concentration of HNO_3_. The adsorption capacity of Re decreased with the increase in the absorbed doses, and the maximum value in 1 M HNO_3_ reached 20.9 mg/g, even with the absorbed dose up to 100 kGy. The decontamination efficiency of ^99^Tc by N3/SiO_2_ was estimated to be 64.5% after adsorption in radioactive waste at 1 M HNO_3_. Hence, N3/SiO_2_ is considered to be a competent and feasible adsorbent and has potential application prospects for the extraction of ^99^Tc from HLLW.

## Figures and Tables

**Table 1:** The pore size and porosity of SiO_2_ and N3/SiO_2_.

Parameters	Average Pore Size (nm)	Porosity (%)
SiO_2_	43.3	75.6
N3/SiO_2_	34.8	70.7

**Table 2:** The estimated isotherm parameters of the adsorption isotherms.

Models	Parameters	Fresh (1 M HNO_3_)	Fresh (1 M HNO_3_–1 M NaNO_3_)	50 kGy (1 M HNO_3_)	100 kGy (1 M HNO_3_)
Langmuir	*Q*_m_ (mg/g)	35.8	22.2	31.9	20.9
	*K*_L_ (L/mg)	0.1022	0.0834	0.1357	0.1918
	*R* ^2^	0.9879	0.9850	0.9936	0.9717
Freundlich	*K*_F_ (mg/g)	3.6	1.8	4.1	3.6
	1/*n*	0.7307	0.8054	0.7241	0.6548
	*R* ^2^	0.9913	0.9804	0.9870	0.9660

## Data Availability

Not applicable.
